# Human skin CD141^+^ dendritic cells regulate cutaneous immunity via the neuropeptide urocortin 2

**DOI:** 10.1016/j.isci.2023.108029

**Published:** 2023-09-26

**Authors:** Prudence PokWai Lui, Chrysanthi Ainali, Chung-Ching Chu, Manuela Terranova-Barberio, Panagiotis Karagiannis, Angela Tewari, Niloufar Safinia, Ehsan Sharif-Paghaleh, Sophia Tsoka, Grzegorz Woszczek, Paola Di Meglio, Giovanna Lombardi, Antony R. Young, Frank O. Nestle, Niwa Ali

**Affiliations:** 1 Peter Gorer Department of Immunobiology, School of Immunology and Microbial Science, King’s College London, London, UK; 2 Centre for Gene Therapy and Regenerative Medicine, School of Basic and Biomedical Sciences, King’s College London, London, UK; 3 St. John’s Institute of Dermatology, King’s College London and NIHR Biomedical Research Centre, London, UK; 4 Institute of Liver Studies, Department of Inflammation Biology, School of Immunology and Microbial Sciences, James Black Centre, King’s College London, London, UK; 5 Department of Imaging Chemistry & Biology, School of Biomedical Engineering & Imaging Sciences, Faculty of Life Sciences & Medicine, King’s College London, London, UK; 6 Department of Informatics, Faculty of Natural, Mathematical and Engineering Sciences, King’s College London, Bush House, London, UK; 7 Asthma UK Centre in Allergic Mechanisms of Asthma, School of Immunology and Microbial Sciences, King’s College London, London, UK

**Keywords:** Immunology

## Abstract

Skin immune homeostasis is a multi-faceted process where dermal dendritic cells (DDCs) are key in orchestrating responses to environmental stressors. We have previously identified CD141^+^CD14^+^ DDCs as a skin-resident immunoregulatory population that is vitamin-D_3_ (VitD3) inducible from monocyte-derived DCs (moDCs), termed CD141^hi^ VitD3 moDCs. We demonstrate that CD141^+^ DDCs and CD141^hi^ VitD3 moDCs share key immunological features including cell surface markers, reduced T cell stimulation, IL-10 production, and a common transcriptomic signature. Bioinformatic analysis identified the neuroactive ligand receptor pathway and the neuropeptide, urocortin 2 (UCN2), as a potential immunoregulatory candidate molecule. Incubation with VitD3 upregulated UCN2 in CD141^+^ DCs and UVB irradiation induced UCN2 in CD141^+^ DCs in healthy skin *in vivo.* Notably, CD141^+^ DDC generation of suppressive Tregs was dependent upon the UCN2 pathway as *in vivo* administration of UCN2 reversed skin inflammation in humanized mice. We propose the neuropeptide UCN2 as a novel skin DC-derived immunoregulatory mediator with a potential role in UVB and VitD3-dependent skin immune homeostasis.

## Highlights

• CD141^+^ skin DDCs and VitD3 moDCs are functionally and transcriptomically similar
• CD141^+^ DDCs and CD141^hi^ VitD3 moDCs preferentially express the neuropeptide UCN2
• Solar UVB irradiation and VitD3 induces UCN2 in CD141^+^ DDCs and moDCs, respectively
• UCN2 protects from alloimmune cell-mediated human skin inflammation *in vivo*

## Graphical abstract

**Figure:**
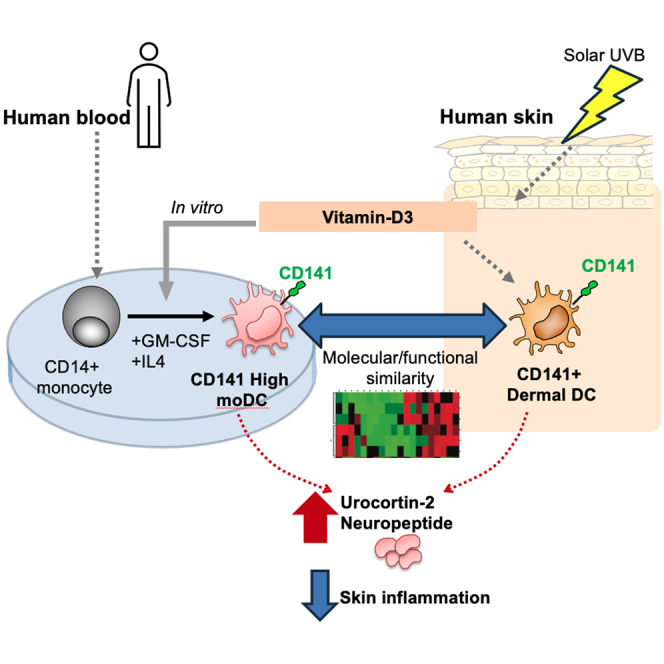

## Introduction

Immune regulation at tissue sites, such as the skin, is important to maintain homeostatic balance in the steady state.^1^^,^^2^ Tissue-residing dendritic cells (DCs) are specialized sentinels of the skin-associated immune system that serve to bridge the gap between innate and adaptive immunity. Upon encountering antigen in the periphery, DCs migrate to T cell-rich areas of lymphoid organs for presentation to T cells bearing antigen-specific receptors. In the absence of peripheral DC activation, steady-state antigen presentation can lead to T cell hyporesponsiveness or tolerance.^3^ This is best demonstrated in murine models of *in vivo* antigen targeting using either DC-specific antibodies^4^ or genetically induced expression of model antigens in DCs^5^ to induce regulatory T cell (Treg)-mediated tolerance. DCs may also play a direct role in Treg maintenance in the peripheral blood via expression of the co-stimulatory receptors CD80 and CD86.^6^ More recently, the capacity to induce self-antigen-specific Treg has been ascribed solely to tissue migratory DCs, and not lymph node-resident DCs.^7^ Taken together, these findings suggest that migratory DCs, or a specific subset thereof, function to continuously sample antigens expressed both in the steady state and upon environmental and/or pathogen-derived stressors to the tissue immune compartment.

One of the most significant environmental factors exerting immunosuppressive effects in human skin is solar UVB radiation (∼295–315 nm). Within this action spectrum, vitamin D is synthesized in the skin and may be converted locally to its immunologically active metabolite, 1a,25-dihydroxyvitamin D3 (VitD3),^8^^,^^9^ of which DCs are the predominant immune cell target.^10^^,^^11^ VitD3 action on DCs induces a regulatory phenotype with reduced co-stimulatory receptor expression and poor effector T cell stimulation. This is usually accompanied with enhanced IL-10 secretion and expansion of Treg, as well as repression of *in vivo* IL-17 producing (Th17) and IFN-g producing (Th1) T helper cells.^12^^,^^13^^,^^14^ In murine models of contact hypersensitivity, sensitization to contact allergen in both solar UV-irradiated skin or in the context of topically applied VitD3 is impaired via expansion of hapten-specific Tregs,^15^^,^^16^ indicating the existence of common regulatory pathways between solar UVB and VitD3. It has been suggested that solar UVB-mediated immunosuppression may be mediated, at least in part, through the action of induced VitD3.^12^ Topical treatment of human skin with VitD3 analogs inhibits the skin immune response to an equivalent extent as solar UVB irradiation.^17^ Furthermore, solar UVB-mediated suppression is abolished in vitamin D receptor knockout mice, indicating a VitD3-dependent mechanism of tolerance induction.^18^ The molecular pathways underlying the cutaneous solar UVB-VitD3 axis, particularly in relation to skin-resident human DC populations are currently unknown.

We have previously identified a population of human dermal DCs (DDCs) resident in human skin, characterized by constitutive IL-10 secretion, ability to migrate to skin draining lymph nodes, and surface co-expression of CD141 and CD14.^19^ VitD3 treatment of blood-derived CD1c^+^ DCs or monocyte-derived (mo)DCs induced CD141^+^CD14^+^ DDC-like functional counterparts. We were able to generate sufficient cell numbers of the CD141^hi^ expressing fraction of VitD3 moDCs to induce alloimmune tolerance in humanized mouse models of disease, demonstrating their potential use in adoptive regulatory DC therapy.^19^

Here, using transcriptome profiling, we identify a shared gene signature of human skin-resident and VitD3-inducible regulatory CD141^+^ DCs and establish the solar UVB and VitD3-inducible neuropeptide, urocortin 2 (UCN2), as a key skin DC-derived immune suppressive mediator with a potential role in cutaneous immune homeostasis.

## Results

### Skin-resident CD141^+^ DDCs and CD141^hi^ VitD3 moDCs share phenotypic and functional similarity

**Figure 1:**
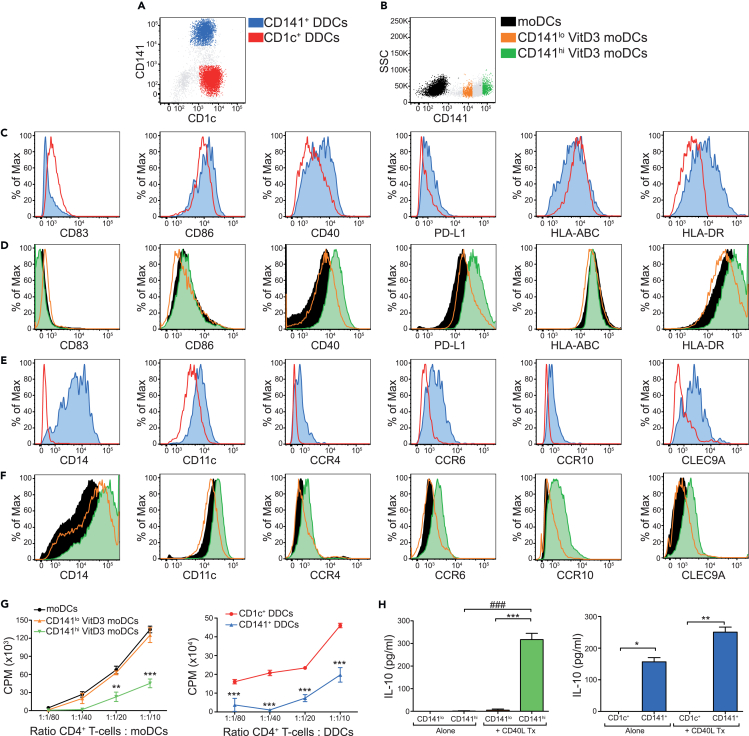
CD141 expressing skin migratory DDCs and VitD3 moDCs share phenotypical and functional characteristics (A) The two major populations of resident DDCs in healthy human skin are CD141^hi^CD1c^lo^ (shown in blue); and CD1c^hi^CD141^–^ (shown in red). (B) moDCs (shown in black) are largely negative for CD141 while VitD3 treatment upregulates its expression; CD141^lo^ VitD3 moDCs (shown in orange) and CD141^hi^ VitD3 moDCs (shown in green). (C‒F) DDC and moDC populations were stained for (C, D) CD83, CD86, CD40, PD-L1, HLA-ABC, HLA-DR, and (E, F) CD14, CD11c, CCR4, CCR6, CCR10, and CLEC9A. (G) moDCs, CD141^lo^ VitD3 moDCs, and CD141^hi^ VitD3 moDCs (left panel); CD141^+^ and CD1c^+^ DDCs (right panel) stimulation of allogeneic CD4^+^ T cell proliferation, measured ascounts per minute (CPM). (H) CD141^lo^ VitD3 moDCs and CD141^hi^ VitD3 moDCs (left panel); CD141^+^ and CD1c^+^ DDCs (right panel) production of IL-10 in the absence and presence of CD40 ligand-transfected L cells (CD40L Tx). Results are representative of (A, B) more than twenty, (C‒F) three, (G) ten, and (H) five independent experiments. Data are represented as mean ± SEM. Two-way ANOVA (G) or one-way ANOVA (H), ∗p < 0.05, ∗∗p < 0.01, ∗∗∗p < 0.001, ^###^p < 0.001. See also Figure S1.

As specialist antigen-presenting immune cells, DCs have the ability to present antigens to T cells and program immune function through multiple mechanisms including signal strength, co-stimulatory molecules, and cytokines. We have previously shown that human CD141^+^ DDCs isolated from skin, or generated from moDCs by treatment with VitD3, induce allogeneic CD4^+^ T cell hyporesponsiveness.^19^ We performed extensive DC immunophenotyping in CD141^+^ DDCs isolated from skin or generated from moDCs by treatment with VitD3 to examine their potential for antigen presentation, co-stimulation, and tissue homing. DDCs were subdivided into CD141^+^ (CD1c^low^ CD141^+^) and conventional CD1c^+^ (CD1c^hi^ CD141^-^) cells (Figure 1A; Full gating strategies are shown in Figure S1A) and co-stained for an array of surface markers. CD141^+^ DDCs express high levels of CD14 and CD11c; they also express higher levels of the co-stimulatory molecule CD40, and of the co-inhibitory molecule PD-L1 as compared to CD1c^+^ DDCs (Figure 1C). While the expression of HLA-ABC (major histocompatibility complex (MHC) class I) is similar between all DC subsets, HLA-DR (MHC class II) is markedly higher on CD141^+^ DDCs and CD141^hi^ VitD3 moDCs (Figures 1C and 1D). Surface expression of the skin homing chemokine receptors CCR4, CCR6, and CCR10^20^ is consistently higher on CD141^+^ than on CD1c^+^ DDCs (Figure 1E). Among the skin migratory DDC population, CD141^+^ DDCs uniquely express CLEC9A (Figure 1E), thought to be an essential molecular marker for MHC class I-restricted cross-presentation of exogenous antigen.^21^^,^^22^^,^^23^ The origin of CD141-expressing cells in this study as myeloid DCs is further validated by their increased cell surface expression of CLEC9A^24^ (Figures 1E and 1F) and complements our previous data showing CLEC9A mRNA expression in sorted CD141^+^ DDCs.^19^ The immune phenotyping mentioned previously was extended to moDCs and CD141^hi^ and CD141^low^ VitD3 moDCs (gated as shown in Figures 1B, S1B, and S1C). With the exception of CD14 that was expressed at intermediate to high levels on all moDCs (Figure 1F), CD141^low^ VitD3-treated moDCs were found to express similar markers to moDCs, and reflected the phenotype seen in CD1c^+^ DDCs, i.e., intermediate expression of CD11c, CD40, and PD-L1 (Figures 1D and 1F), low levels of the chemokine receptors CCR4, CCR6, and CCR10, and low levels of the cross-presentation-associated molecule CLEC9A (Figure 1E). Conversely, CD141^hi^ VitD3 moDCs mirrored the phenotype seen on CD141^+^ DDCs, i.e., CD14^hi^, CD40^hi^, PD-L1^hi^, CD11c^hi^, and clear expression of CCR6, CCR10, and CLEC9A (Figures 1D and 1F).

In keeping with their similar cell surface phenotype, CD141^hi^ VitD3 moDCs, like their CD141^+^ DDC skin counterparts,^19^ were poor stimulators of allogeneic T cells and produced high levels of IL-10 following ligation of CD40 (Figures 1G and 1H). CD141^lo^ VitD3 moDCs on the other hand were able to stimulate robust allogeneic CD4 T cell proliferation and produced negligible amounts of IL-10 either in the steady state or following CD40L stimulation. Immature moDCs generated in the absence of VitD3 were similar to CD141^lo^ VitD3 moDCs (Figures 1G and 1H). Overall, we demonstrate that skin-resident CD141^+^ DDCs share striking similarity in both phenotype and function with CD141^hi^ VitD3 moDCs, but not CD141^lo^ VitD3 moDCs or moDCs, implicating the presence of a VitD3-responsive moDC precursor population in the periphery defined by high expression of CD141.

### CD141^+^ DDCs and CD141^hi^ VitD3 moDCs share a common transcriptomic signature

**Figure 2:**
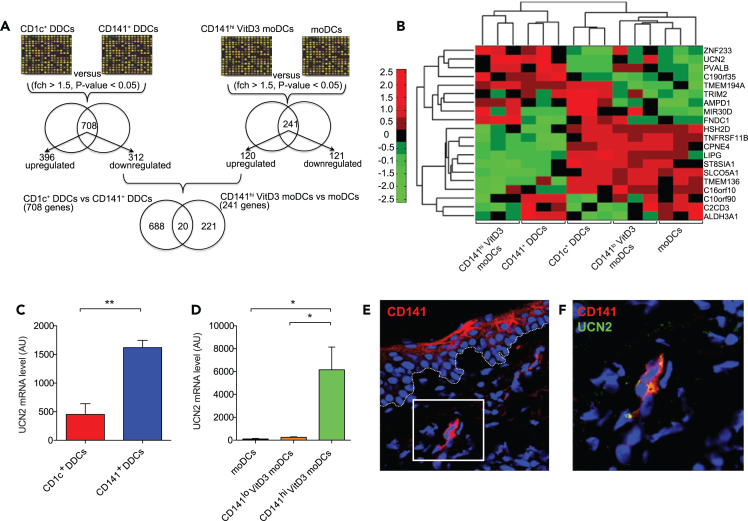
CD141^+^ DDCs and CD141^hi^ VitD3 moDCs share a common transcriptomic profile and preferential expression of the neuropeptide UCN2 (A) Pairwise strategy to investigate similarities in the transcriptomes of skin-resident CD141^+^ DDCs and CD141^hi^ VitD3 moDCs. (B) Identification of common differentially expressed genes between CD1c^+^ DDCs/CD141^+^ DDCs and CD141^hi^ VitD3 moDCs/moDCs are summarized in a heatmap, and subjected to bidirectional hierarchical clustering. (C) The differential expression of UCN2 was validated in DDC and moDC populations by qPCR. (D) CD141 immunofluorescence staining (red) co-localizes with UCN2 (green; right panel) in healthy human skin, indicating steady-state expression *in situ*. Error bars indicate SEM. Results are combined data of (B) three and (C, D) five human blood and skin donors. (E) Representative of five human skin donors. Data are represented as mean ± SEM. Unpaired t test (C), one-way ANOVA (D), ∗p < 0.05, ∗∗p < 0.01. See also Tables S1 and S2, and Figure S2.

To investigate the molecular basis of the similarity in phenotype and function of skin CD141^+^ DDCs and blood CD141^hi^ VitD3 moDCs, we took an unbiased discovery approach to identify pathways involved in their immunoregulatory function by studying global gene expression. We firstly analyzed the expression of mRNA encoding ZBTB46 (zDC), a recently identified transcription factor specific for the classical DC lineage both in humans and mice.^25^^,^^26^ We found that all skin and moDC populations expressed equal levels (Figure S2A). To determine the common immunoregulatory pathways active in skin-resident DDCs and VitD3 moDCs, we designed a strategy to compare mRNA expression in a pairwise fashion between CD1c^+^ DDCs/CD141^+^ DDCs and CD141^hi^ VitD3 moDCs/moDCs (Figure 2A). We applied a cutoff of p < 0.05 and fold-change greater than 1.5 to determine genes that were differentially expressed. Differential gene expression of CD141^+^ DDCs versus CD1c^+^ DDCs defined a molecular signature of 708 genes, of which 396 showed an overexpression in CD141^+^ DDCs and the remaining 312 genes underexpressed. Differential expression of CD141^hi^ VitD3 moDCs versus moDCs established a molecular signature of 241 genes, 120 being overexpressed in CD141^hi^ VitD3 moDCs, the remaining genes exhibiting lower expression (Figure 2A; Tables S1 and S2). Transcriptome analysis of CD141^+^ DDCs and CD141^hi^ VitD3 moDCs identified a common molecular signature, including a core module of 20 common differentially expressed genes. To analyze functional similarity between DC subsets, we used a hierarchical clustering approach which demonstrated that CD141^+^ DDCs clustered together with CD141^hi^ VitD3 moDCs, indicating a molecular relationship (Figure 2B). We also selected differentially expressed genes from the core module (i.e., up- and downregulated in all DC populations) and confirmed their expression levels via qPCR. Gene expression levels correlated with the intensities generated from our transcriptome arrays (Figures S2B‒S2E), thus validating the array platform and our analysis strategy. Our data show that skin CD141^+^ DDCs and blood CD141^hi^ VitD3 moDCs share a common module of gene expression, suggesting the existence of common pathways involved in their regulatory function.

### The neuropeptide UCN2 and UCN2 pathway activity are overexpressed in CD141^+^ DDCs and CD141^hi^ VitD3 moDCs

We next analyzed the global gene transcripts for biological pathway activity to determine common factors associated with skin-resident and VitD3-generated CD141^+^ DCs (see STAR Methods and Figure S3 for pathway analysis strategy). To quantify the activity per pathway, a *Z* score scale (dimensionless quantity) was used to show the degree of similarity of one population (DC subset) to another. DC subsets with large positive or negative *Z* score activity for a pathway differ strongly from the reference DC subset (CD141^+^ DDCs) for the pathway analyzed. Conversely, DC subsets with a mean *Z* score pathway activity value close to 0 show similar phenotypic patterns with the corresponding pathway in the reference subset. Comparison of the molecular pathways associated with CD141^+^ DDCs and CD141^hi^ VitD3 moDCs demonstrated that the most similar *Z* scores between the two populations were in the neuroactive signaling pathway, while the *Z* scores of CD1c^+^ DDCs, CD141^lo^ VitD3 moDCs, and moDCs were more distant from 0 (Figure S2F). This pathway includes the interaction of a neuropeptide, UCN2, with its cognate receptor corticotrophin-releasing hormone receptor 2 (CRHR2).^27^ Indeed, UCN2 appeared as one of the twenty genes that were commonly differentially expressed in the hierarchical clustering analysis (shown in Figure 2B). To validate the differential expression of UCN2, we assessed mRNA in the DC populations by qPCR. Indeed, CD141^+^ DDCs and CD141^hi^ VitD3 moDCs expressed significantly higher mRNA levels than CD1c^+^ DDCs, CD141^lo^ VitD3 moDCs, and moDCs (Figures 2C and 2D). We next sought to confirm *in situ* UCN2 protein expression by resident CD141^+^ DDCs using immunofluorescence histology of healthy human skin. CD141^+^ DDCs were positive for UCN2 in the steady state, indicating constitutive expression of the neuropeptide *in vivo* (Figures 2E and 2F). In line with previous findings, CD141 staining could also be seen in epidermal keratinocytes.^28^ These findings raise the question whether the neuropeptide UCN2 and its associated neuroactive pathway could potentially be involved in the immunoregulatory function of CD141^+^ DCs.

### UCN2 is inducible in CD141^+^ DCs by VitD3 *in vitro*, and in UVB-irradiated human healthy skin *in vivo*

**Figure 3:**
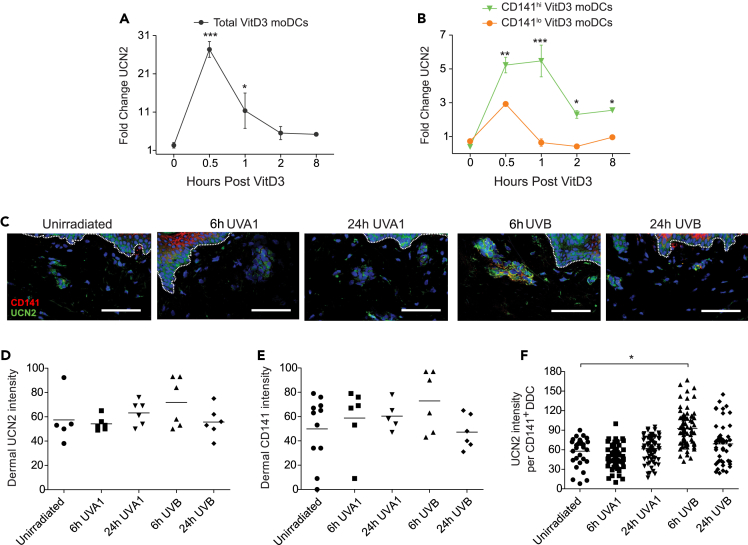
Solar UVB and VitD3 induce UCN2 in CD141^hi^ VitD3 moDCs and CD141^+^ DDCs *in vivo* (A and B) Total VitD3-treated moDCs, or (B) FACS-sorted CD141^lo^ VitD3 moDCs, and CD141^hi^ VitD3 moDCs at time intervals post VitD3 treatment and assessed for UCN2 expression via qPCR. (C) Human skin was biopsied from healthy volunteers prior to irradiation and at 6 and 24 h post irradiation with 1 MED of either 50 J/cm^2^ UVA1 or 30 mJ/cm^2^ solar UVB and assessed via immunofluorescence for CD141 and UCN2 expression. Bars: 100μm. (D and E) The total dermal UCN2 staining intensity and (E) dermal CD141 staining intensity was quantified. (F) The intensity of UCN2 staining was quantified per CD141^+^ DDC as a measure of inducibility on a per cell basis *in situ*. Error bars indicate SEM. (A, B) Data are combined from three human blood donors from three independent experiments; representative (C) or combined (D–F) from three healthy volunteers in total. Data are represented as mean ± SEM. two-way ANOVA (B), or one-way ANOVA (A, F), ∗p < 0.05, ∗∗p < 0.01, ∗∗∗p < 0.001. See also Figures S4 and S5.

Since UCN2 is only weakly expressed in moDCs and CD141^lo^ VitD3 moDCs but is highly expressed by CD141^hi^ VitD3 moDCs (Figure 2D), we hypothesized that UCN2 may be upregulated by VitD3. We first tested the kinetics of UCN2 induction in the total population of moDCs and found its expression was rapidly upregulated following addition of VitD3 (Figure 3A). To better understand if the source of this increase was derived from a VitD3-responsive moDC subpopulation, we sorted CD141^hi^ and CD141^lo^ moDCs at defined time course intervals post VitD3 treatment. Indeed, the immunoregulatory CD141^hi^ fraction were the major producers of UCN2, with a 5-fold induction of expression at 1 h post addition of VitD3 compared to a more modest and short-lived induction in CD141^lo^ moDCs (Figure 3B). We then sought to translate this finding of VitD3 responsiveness *in vitro* to *in vivo* human skin biology.

DDCs are ideally placed to respond to VitD3, which is readily induced by solar UVB radiation (∼295–315 nm). To test whether UCN2 peptide could be induced *in vivo*, we recruited healthy human volunteers into a study where a defined portion of lower back skin was exposed to a single dose (approximately 1 minimal erythemal dose, MED) of either 50 J/cm^2^ UVA1 (340–400 nm) or 30 mJ/cm^2^ solar UVB (300 nm) radiation (Table 1).^29^ 300 nm is about the peak of the action spectrum for the formation of vitamin D.^30^^,^^31^ Biopsies were taken prior to irradiation and at 6 and 24 h post irradiation and tissue sections were assessed via immunofluorescent co-staining for UCN2 and CD141 (Figure 3C). The overall intensity of UCN2 and CD141 expression in the dermis was not significantly changed by either UVA1 or solar UVB radiation (Figures 3D and 3E). However, the intensity of UCN2 staining on or around CD141^+^ DDCs was significantly increased at 6 h after UVB but not UVA1 radiation (Figure 3F), indicating an upregulation of UCN2 in the presence of solar UVB-induced VitD3 synthesis *in vivo*. Given the overall induction of UCN2 measures the intensity for the entire dermis (Figure 3D), the specific upregulation of UCN2 in CD141+ DCs is likely diluted when assessing the entire pool of dermal-resident cells. Previous studies have demonstrated expansion of CD141+ DCs in human skin following UVB irradiation at approximately 2 MED.^32^ While our study utilizes a slightly less inflammatory UVB dose equivalent to 1 MED, we observe a consistent accumulation of CD141+ DCs at 6 h after UVB exposure, which then declines at 24 h approaching baseline levels (Figure S4). The expansion of CD141+ cells solidifies the importance of these cells in response to cutaneous UVB irradiation.

**Table 1:** Healthy volunteer participants

	Skin type	Sex	Age	MED UVA1 (J/cm^2^)	MED solar UVB (mJ/cm^2^)
	II	F	24	61.1	37.0
	II	F	27	61.1	37.0
	I	F	22	48.8	30
**Mean ± SD**			**24.3 ± 2.5**	**57.0 ± 7.1**	**34.7 ± 4.0**

Given that *in situ* upregulation of UCN2 in skin-residing CD141+ cells peaks at 6 h and starts to decline 24 h post UVB exposure, we next sought to determine if induction of UCN2 in VitD3-induced CD141-high moDCs is similarly transient. The expression of UCN2 persisted at equivalent levels for all time points tested until at least 120 h (5 days) post VitD3 exposure (Figure S5), indicating CD141-high moDCs can retain immunosuppressive identity for extended time periods. Taken together, the induction of UCN2 by solar UVB at a dose which is optimal for VitD3 synthesis suggests the involvement of VitD3 in solar UVB-mediated upregulation of UCN2 in CD141^+^ DDCs.

### UCN2 is required for Treg function and protects from inflammatory skin pathology *in vivo*

**Figure 4:**
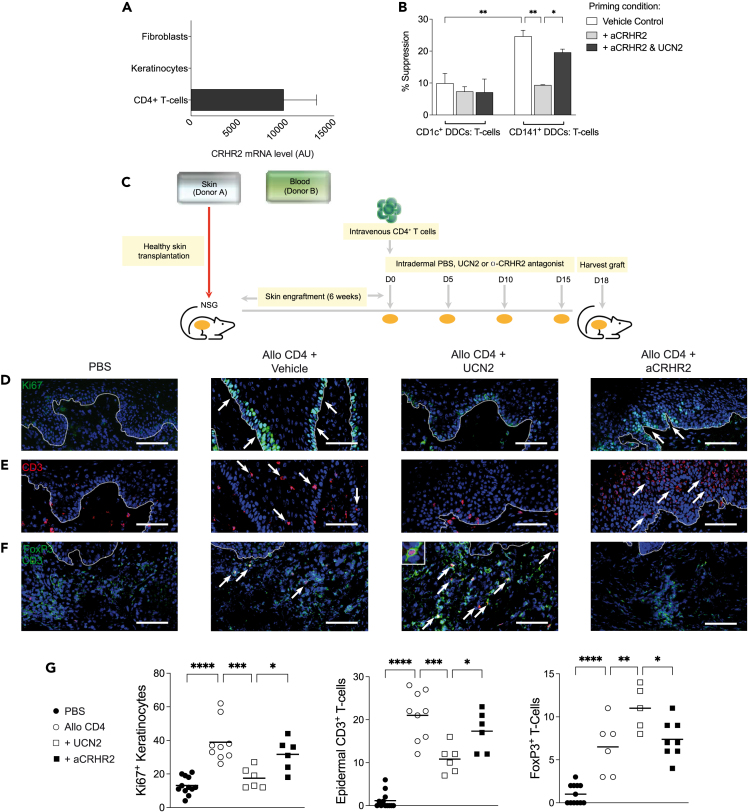
UCN2 protects from alloimmune cell-mediated human skin inflammation *in vivo* (A) qPCR CRHR2 expression in cell populations isolated from human skin and blood, presented as arbitrary units (AU). (B) T cell suppression assay from sorted CD25^hi^ T cells from co-cultures with either CD1c^+^ DDCs or CD141^+^ DDCs in the presence of vehicle control, CRHR2 antagonist (aCRHR2) alone, or together with UCN2 peptide. (C‒F) Experimental strategy to assess contribution of intradermally injected vehicle (PBS), aCRHR2 or UCN2 into human skin grafted NSG mice receiving intravenous PBS or allogeneic CD4^+^ T cells. Representative fields from stained skin grafts for (D) epidermal keratinocyte expression of the proliferation marker Ki67 (arrows), (E) epidermal CD3^+^ T cell infiltration (arrows), and (F) dermal FoxP3^+^CD3^+^ T cells (arrows). Dashed lines indicate epidermal-dermal junction. Bars: 100μm. (G) Quantitative histological analysis of at least three independent visual fields per skin graft. Lines represent the mean ± SEM. (n = 3–6 animals per treatment group). Results are combined data from two independent experiments. One-way ANOVA test (B, G), ∗p < 0.05, ∗∗p < 0.01, ∗∗∗p < 0.001 See also Figure S6.

We next asked whether UCN2 has an immunoregulatory role in maintaining skin homeostasis through blockade of skin inflammation. To better understand the consequence and potential paracrine action of UCN2 secretion by CD141^+^ DDCs, we firstly analyzed the expression of the specific receptor for UCN2, CRHR2,^27^ by qPCR on dermal accessory cells (fibroblasts), epithelial cells (keratinocytes), and the major interacting T cell partner – peripheral CD4^+^ T cells.^19^ Expression of CRHR2 was detected at appreciable levels only in CD4^+^ T cells (Figure 4A).

Since UCN2 is expressed by immunoregulatory CD141^+^ DC subsets and CD4^+^ T cells express CRHR2, we next hypothesized that the interaction of UCN2 and CRHR2 could be involved in Treg generation and/or function. We first tested if UCN2 can act directly on CD4^+^ T cells via supplementation with UCN2 peptide. Flow cytometric analysis revealed no changes in the proportion and per cell expression intensity of Ki67 or the major Treg activation markers CTLA4, ICOS, and CD25 either in the presence or absence of UCN2. Similarly, transcript for CTLA-4 and the lineage-defining transcription factor, FoxP3, were unchanged between conditions (Figure S6), suggesting UCN2 alone is not sufficient to drive the expression of Treg-associated markers in CD4^+^ T cells.

Given we have shown previously that CD4^+^CD25^hi^ T cells generated by stimulation with CD141^+^ DDCs are more suppressive than those generated from CD1c^+^ DDCs,^19^ we next sought to test the involvement of UCN2 in this experimental scenario. We first sorted CD25^hi^ T cells generated by co-culture with either CD141^+^ or CD1c^+^ DDCs, and tested their ability to suppress a polyclonal CD4^+^ T cell response. The regulatory function of CD141^+^ DDCs, but not CD1c^+^ DDCs, was dependent on UCN2 signaling since CD25^hi^ T cells generated in the presence of the specific CRHR2 peptide antagonist (aCRHR2)^27^ abrogated their ability to supress T cell proliferation (Figure 4B). This effect could be overcome and partially restored to basal proliferative levels in CD141^+^ DDC co-cultures by adding UCN2, confirming the importance of UCN2 in the generation of functionally suppressive Tregs by CD141^+^ DDCs.

In order to test the immunoregulatory potential of UCN2 *in vivo*, we took advantage of a previously established humanized mouse model of inflammatory skin pathology in which allogeneic T cell responses can be inhibited by CD141^+^ DCs or by Tregs generated from CD141^+^ DDCs.^19^ Recipient immunodeficient NOD-*scid* IL-2Rγ^null^ mice were transplanted with healthy human skin and allowed to heal over a six-week period. One group of mice received only allogeneic CD4^+^ T cells to induce an inflammatory anti-human skin graft response, while other groups received additional intra-dermal injections of either UCN2, aCRHR2, or PBS directly into the human skin graft (Figure 4C). Mice were sacrificed 18 days after infusion of CD4^+^ T cells and the skin grafts were examined histologically for signs of alloimmune inflammatory skin pathology.^19^^,^^33^ Mice which had not received CD4^+^ T cells showed basal turnover of keratinocytes of 10–20 cells/field in the basement membrane while those that received allogeneic T cells showed clear signs of inflammatory skin pathology including increased turnover of keratinocytes and significant epidermal T cell infiltration (Figures 4D, 4E, and 4G). Intradermal injection of UCN2 was able to reverse the allogeneic T cell-mediated inflammatory response, as indicated by the return of Ki67 staining of keratinocytes to almost basal levels of proliferation and significant reduction of T cell infiltration into the epidermis (Figures 4D, 4E, and 4G). Furthermore, dermal T cells in the skin grafts of UCN2-treated mice were largely Foxp3^+^, suggesting the preferential induction or recruitment of Tregs in the presence of exogenous UCN2 (Figures 4F and 4G). In contrast, no significant changes in inflammatory markers or FoxP3^+^ staining were observed in aCRHR2-treated skin grafts (Figures 4D–4G). Overall, we conclude that UCN2 is an anti-inflammatory neuropeptide that is able to reverse human inflammatory skin pathology *in vivo*, and is thus a potential candidate as a locally administered anti-inflammatory cutaneous therapeutic agent.

## Discussion

The identification and validation of functional pathways utilized by tissue-resident immune cells to control inflammation are challenging due to limited access to human tissue sites and the lack of relevant *in vivo* models. While much effort has been directed toward immune cell ontology and phenotyping, the mechanisms of action employed by critical regulators of immune homeostasis, such as skin-resident DCs, are largely unexplored in the context of human disease. Here, we characterize the major human skin-resident dermal DC populations at the molecular and functional *in vivo* level to identify the neuropeptide UCN2 and its pathway as a major regulator of solar UVB mediated skin immune homeostasis. We demonstrate that skin-resident CD141^+^ DDCs and CD141^hi^ VitD3 moDCs are phenotypically and functionally similar, share a common transcriptomic profile, and preferentially express UCN2. Further, CD141^+^ DC induction of Tregs was dependent on the UCN2 pathway and, when tested in an *in vivo* model of effector cell-mediated human skin inflammation, UCN2 significantly ameliorated tissue pathology.

UCN2 has been identified and characterized as the second mammalian member of the corticotrophin-releasing factor (CRF) family,^27^ a group of evolutionarily conserved peptides with homologs in fish, amphibians, and mammals. It is well established that the CRF signaling system regulates neuroendocrine homeostasis in human skin.^34^ Despite the knowledge that CRF components are expressed in multiple keratinocyte, fibroblast, and circulating immune cells in both mice and humans,^35^ very little is known about the involvement of this pathway in immune regulation, in particular the downstream effects and exact cellular source of UCN2. To assess expression across cell types, we explored the human protein atlas database to query UCN2 expression in human skin. Expression is detected primarily in basal epithelial keratinocyte populations, but also fibroblasts, endothelial, smooth muscle, and macrophage populations. The latter likely correspond most closely to the CD141+ DCs we analyze in our manuscript (https://www.proteinatlas.org/ENSG00000145040-UCN2/single+cell+type/skin). Indeed, consistent with our immunofluorescence staining, UCN2 expression is present in cells other than CD141+ DCs (Figure 3C).

However, there is evidence that the UCN2 pathway can upregulate VitD3 receptor expression in breast cancer as a pro-apoptotic mechanism,^36^ indicating the existence of a positive feedback loop that may also be relevant in the cutaneous environment. Others have also postulated an immune-inhibitory role for UCN2 by showing an acceleration of macrophage apoptosis acting via CRHR2^37^ that was later associated with an inhibition of TNFα release^38^ and increased synthesis of IL-10.^39^ In experimentally induced murine colitis, UCN2 receptor knockout mice (CRHR2^−/−^) show exacerbated intestinal inflammation^40^ and increased mortality.^41^ Similarly, in murine collagenase-induced arthritis, UCN administration can drive *in vivo* expansion of CD4^+^CD25^+^ Tregs in both lymph nodes and within joints.^42^ These findings support a tissue regulatory role for the UCN2 ligand receptor pathway. In murine skin, UVB irradiation can induce expression of the urocortin peptide family in the dermal compartment,^43^ supporting our finding of UCN2 upregulation in dermal-resident CD141^+^ DDCs of solar UVB-irradiated human skin. Recently, human CD141^+^ DDCs themselves have been shown to increase up to 5-fold in number following UVB irradiation,^32^ an effect that is also associated with a significant reduction in the other major resident DDC population, CD1c^+^ DDCs,^44^ suggesting that CD141^+^ immune regulatory DDCs may play a prominent functional role in the response to solar UVB.

The increased expression of PD-L1 in both CD141^+^ DDCs and CD141^hi^ VitD3 moDCs indicates DC populations with the potential to generate inhibitory signals in T cells. Interaction with PD-1 attenuates signaling through the T cell receptor and has been associated with inhibiting cytolytic function and effector cytokine production while promoting the development of peripherally derived Treg.^45^^,^^46^^,^^47^ Notably, DC expression of PD-L1 has been previously associated with VitD3 and Treg induction.^48^ We show here that VitD3 induced the highest levels of PD-L1 in CD141^hi^ VitD3 moDCs. This also holds true for the expression of the skin homing markers CCR4 and CCR10, indicating a VitD3-mediated skin “imprinting” specifically in CD141^hi^ VitD3 moDCs. Indeed, this phenomenon also holds true for T cells exposed to VitD3, which readily upregulate CCR10 to direct migration toward epidermal keratinocytes.^49^

One limitation of our study is the lack of direct clinical evidence that targeting of UCN2 or its pathway can substantially modify on-going skin inflammation in humans. A recent systemic meta-analysis of 42 clinical trials shows that short-term UCN2 infusion can reduce mean arterial pressure and reduced ejection fraction, making it a promising therapy for heart failure.^50^ Recently, UCN2 has been shown to promote skeletal muscle health, and gene transfer of UCN2 results in reduced fatty infiltration of the liver in mice with insulin resistance,^51^ proposing their potential beneficial role in type 2 diabetes treatment. Our data may incite a clinical re-evaluation of the *in vivo* anti-inflammatory effects of UCN2 at other tissue sites and contribute to the development of clinical trials to directly assess the role of UCN2 in treating T cell-mediated cutaneous pathology. In addition, we have identified a clinically transferable approach to enrich immunoregulatory UCN2-producing DCs based on the selection of CD141-high-expressing human moDCs induced by VitD3 treatment. Modulating the immune response accordingly in patients with tissue inflammatory disease using autologous CD141^hi^ VitD3 moDC therapy is technically feasible using Good Manufacturing Practice (GMP) facilities. While many GMP protocols have been established for adoptive Treg cell therapy,^52^ clinical-grade DC isolation and sorting for enriched regulatory populations remains to be fully validated.

Taken together, we show that skin-resident and VitD3-induced CD141^+^ DCs share similarity in expression of key cell surface receptors, IL-10 production, reduced T cell stimulatory capacity, and a common transcriptomic signature. Our *in vivo* experiments further identify a key immunoregulatory mediator, the neuropeptide UCN2, as being inducible in CD141^+^ DCs by both VitD3 and solar UVB irradiation in humans. UCN2 production by CD141^+^ DCs contributes to the generation of functionally suppressive Tregs and was able to suppress allogeneic T cell-mediated skin pathology in a humanized mouse model of tissue inflammation. Overall, this study sheds new light on putative mechanisms of immunoregulatory skin DC subsets, evokes the neuroendocrine-immune axis as a possible mediator of human solar UVB-mediated skin immune homeostasis, and indicates the UCN2 pathway as a therapeutic target in inflammatory skin disease.

### Limitations of the study

While our study identified VitD3 as a potent inducer of CD141 expression in moDCs, whether CD141-high moDCs possess functional skin homing potential when transferred *in vivo* is an important and currently untested question. While these cells express the skin-associated chemokine receptors CCR4, CCR6, and CCR10 (Figure 1F), skin homing capacity could be further investigated upon *in vivo* transfer of CD141-high moDCs in immunodeficient mice xenografted with human skin.

Our findings also indicate that CD141-derived UCN2 impacts the immunosuppressive ability of CD25hi Tregs *in vitro*. Future studies will determine if T cells and UCN2-producing cells are spatially colocalized in skin under conditions of UVB irradiation.

## STAR★Methods

### Key resources table

**Table:** 

REAGENT or RESOURCE	SOURCE	IDENTIFIER
**Antibodies**		

Monoclonal anti-Human CD278 (ICOS)-VioGreen (Clone: REA192)	Miltenyi Biotec	Cat # 130-100-740
Monoclonal anti-Human CD127 - APC (Clone: REA614)	Miltenyi Biotec	Cat #130-113-413
Monoclonal anti-Human CD25 -PE (Clone: REA570)	Miltenyi Biotec	Cat #130-113-286
Monoclonal anti-Human CD45 -VioBright515 (Clone:REA747)	Miltenyi Biotec	Cat #130-110-640
Goat Polyclonal anti-mouse IgG (H+L) AlexaFluor488	Invitrogen	Cat #A-11001
Goat Polyclonal anti-mouse IgG (H+L) AlexaFluor555	Invitrogen	Cat # A21422
Goat Polyclonal anti-rabbit IgG (H+L) AlexaFluor555	Invitrogen	Cat #A-21428
Goat Polyclonal anti-rabbit IgG (H+L) AlexaFluor556	Invitrogen	Cat #A-11008
Mouse monoclonal anti-human CD11c-PerCp/Cy5.5 (Clone: Bu15)	BioLegend	Cat #337209
Mouse monoclonal anti-human CD14-FITC (Clone: TUK4)	Invitrogen	Cat #MHCD1401
Mouse monoclonal anti-human CD141/BDCA3-APC/PE (Clone: AD5-14H12)	Miltenyi Biotec	Cat # 130-113-314
Mouse monoclonal anti-human CD19-FITC (Clone: HIB19)	BD	Cat #560994
Mouse monoclonal anti-human CD194(CCR4)-PECy7 (Clone:1G1)	BD	Cat #561034
Mouse monoclonal anti-human CD196 (CCR6)-PerCp/Cy5.5 (Clone: 11A9)	BD	Cat #560467
Mouse monoclonal anti-human CD1c/BDCA1-PE/APC (Clone: AD5-8E7)	Miltenyi Biotec	Cat # 130-113-302
Mouse monoclonal anti-human CD20-FITC (Clone: L27)	BD	Cat #340673
Mouse monoclonal anti-human CD25-PE (Clone: 2A3)	BD	Cat #341011
Mouse monoclonal anti-human CD3 unconjugated (Clone: F7.2.38)	Aligent Dako	Cat # M7254
Mouse monoclonal anti-human CD3-FITC (Clone: UCHT1)	eBioscience	Cat #11-0038-42
Mouse monoclonal anti-human CD45-PECy7 (Clone: H130)	eBioscience	Cat # 25-0459-42
Mouse monoclonal anti-human CD56-FITC (Clone: NCAM16.2)	BD	Cat #340410
Mouse monoclonal anti-human CD83-FITC (Clone: HB15e)	Invitrogen	Cat #MHCD8301
Mouse monoclonal anti-human CD86-FITC (Clone: BU63)	Invitrogen	Cat #MA1-10295
Mouse monoclonal anti-human CLEC9A-PE (Clone: 8F9)	Miltenyi Biotec	Cat# 130-097-368
Mouse monoclonal anti-human HLA-ABC-PE (Clone: DX17)	BD	Cat #560168
Mouse monoclonal CD40-FITC (Clone: HB14)	Invitrogen	Cat #CD4001
Mouse monoclonal HLA-DR-PE (Clone: TU36)	Invitrogen	Cat # MHLDR04
Mouse monocolonal anti-Human CD4 -BV785 (Clone: OKT4)	BioLegend	Cat #317442
Mouse monocolonal anti-Human CD8a - BV650 (Clone: RPA-T8)	BioLegend	Cat #301042
Mouse monocolonal anti-Human CTLA-4 - BV605 (Clone: BNI3)	BioLegend	Cat #369610
Mouse monocolonal anti-Human FoxP3 - BV421 (Clone: 206D)	BioLegend	Cat #320124
Mouse monocolonal anti-Human Ki-67 - BV711 (Clone: Ki-67)	BioLegend	Cat #350516
Mouse monocolonal anti-Human CD3 - BUV395 (Clone: UCHT1)	BD Biosciences	Cat #563546
Purified anti-mouse anti-human Ly6G/Ly6C (Gr1) mAb (Clone: RB6-8C5)	BioXell	Cat # BE0075
Rabbit monoclonal anti-human Ki-67 unconjugated (Clone: BLR021E)	Abcam	Cat # ab243878
Rabbit polyclonal anti-human Urocortin 2	Phoenix Pharmaceuticals	Cat #G-019-30
Rat monoclonal anti-human CCR10-PE (Clone:314305)	R&D Systems	Cat #FAB3478P-025

**Biological samples**		

Human whole blood	NBS Tooting, NCI London	Cat #NC13
Human whole blood	Donors recruited under ethics 06/Q0704/18	N/A
Human Leukocyte cones	NBS Tooting, NCI London	Cat #NC24
Human skin	Donors recruited under ethics 06/Q0704/18	N/A

**Chemicals, peptides, and recombinant proteins**		

[H^3^] thymidine	Amersham Bioscience	Discontinued
1α,25-Dihydroxyvitamin D3, ≥99% (HPLC)	Sigma	Cat #D1530-10UG
500ml Lymphoprep™	StemCell	Cat #07851
ACK Lysing Buffer	ThermoFIsher	Cat# A10492-01
Animal-Free Recombinant Human GM-CSF	Peprotech	Cat #AF-300-03
Anti-sauvagine-30 (anti-CRHR2 antagonist)	Phoenix Pharmaceuticals	Cat #019-25
ArC™ Amine Reactive Compensation Bead Kit (for use with LIVE/DEAD™ Fixable dead cell stain kits)	ThermoFisher	Cat #A10346
BD CellFix (10x Concentration )	BD	Cat #340181
Bioanalyser RNA Nano and Pico kits	Agilent Technologies	Cat #5067-1511 & #5067-1513
DAPI	Invitrogen	Cat #D1306
Dispase, animal carrier free	StemCell	Cat #07446
Dynabeads™ Mouse T-Activator CD3/CD28 for T-cells	Invitrogen	Cat #11456D
eBioscience™ Foxp3 / Transcription Factor Staining Buffer Set	ThermoFisher	Cat #00-5523-00
Fetal Bovine Serum	Gibco	Cat# A5209
HEPES (1M), Quantity: 100mL	Gibco	Cat# 12509079
Human FAM-CTLA4 XS TaqMan probe	ThermoFisher	Cat #4453320 (Hs00175480_m1)
Human FAM-Foxp3 XS TaqMan probe	ThermoFisher	Cat #4453320 (Hs01085834_m1)
Human FAM-GAPDH XS TaqMan probe	ThermoFisher	Cat #4453320 (Hs02786624_g1)
Human FAM-LIPG, S 250rxns	ThermoFisher	Cat #4331182 (Hs00195812_m1)
Human FAM-PVALB, S 250rxns	ThermoFisher	Cat #4331182 (Hs00161045_m1)
Human IL-4, research grade, 10ug	Miltenyi Biotech	Cat #130-095-373
Human serum, from human male AB plasma, USA origin, sterile -filtered	Sigma-Aldrich	Cat #H4522-20ML
Human UCN2 FAM-MGB, S 250 rxns	ThermoFisher	Cat #4331182 (Hs00264218_s1)
Human Urocortin II peptide 0.5mg	Bachem	Cat #4040984
Human Urocortin II peptide	Phoenix Pharmaceuticals	Cat #019-30
Human VIC-GAPDH, S 360 rxns	ThermoFisher	Cat #4448489 (Hs02786624_g1)
iScript™ cDNA Synthesis Kit, 100 x 20 μl rxns	BioRad	Cat# 1708891
Penicillin/ streptomycin (5000U/ml)	Gibco/Invitrogen	Cat #15070063
ProLong Gold antifade reagent with DAPI	Invitrogen	Cat #P36931
Rainbow Calibration Particles (8 peaks), 3.0 - 3.4 μm	BD	Cat #559123
Recombinant Human IL-4 Protein, Carrier Free	R&D system	Cat #204-IL-020/CF
RosetteSepTM Human CD4^+^ Enrichment Cocktail, 200ml whole blood	StemCell	Cat #TR8300-A
Rosettesep™ Human Monocyte Enrichment Cocktail	Stem Cell	Cat #15068
RPMI 1640 (supplemented with L-glutamine)	Gibco	Cat #11875101
RPMI 1640, no phenol red	Gibco	Cat #11835063
Single donor plasma	NBS Tooting, NCI London	Cat #NC05
TaqMan PreAmp Master Mix Kit-40 reactions	ThermoFisher	Cat# 4384267
UltraComp eBeads Compensation Beads-100 tests	ThermoFisher	Cat# 01-2222-42
Zombie UV Fixable Viability kit	BioLegend	Cat #423108
Superscript II Reverse Transcriptase	Invitrogen	Cat # 18064022
CD25 microbead II, human	Miltenyi biotech	Cat #130-092-983

**Critical commercial assays**		

MILLIPLEX MAP Human Cytokine/Chemokine Magnetic Bead Panel - Immunology Multiplex Assay	Merck Millipore	Cat #HCYTOMAG-60K (HCYIL10-MAG)
Macherey-Nagel NucleoSpin RNA XS Column, Format: Mini spin column	Macherey-Nagel	Cat# 41105518
TargetAmp™-Nano Labeling Kit for Illumina® Expression BeadChip®	Epicentre	Cat #TAN091096
Human Ref-6 Expression BeadChips	Illumina, Ambion	Discontinued

**Deposited data**		

Non-normalised and analyzed data	This paper	Database: GSE243690

**Experimental models: Cell lines**		

CD40 ligand transfected L cells (CD40L Tx)	Provided by Prof. Giovanna Lombardi	N/A

**Experimental models: Organisms/strains**		

NOD/scid/*IL-2Rγ*^*-/-*^ mice (NOD.cg-Prkdc^scid^Il2rg^tm1Wjl^/SzJ)	Jackson Laboratory	Cat # **005557**

**Software and algorithms**		

FlowJo	FlowJo LLC	https://www.flowjo.com/
MATLAB software (functions from Bioinformatics Toolbox)	MathWorks	https://uk.mathworks.com/products/matlab.html (http://www.mathworks.com/help/toolbox/bioinfo/)
DAVID 6.7 bioinformatics database	National Cancer Institute	http://david.abcc.ncifcrf.gov/
Molecular Signature Database (MSigDB database v3.0, downloaded in September 2010)	Subramanian et al.^53^ , Liberzon et al.^54^ & Mootha et al.^55^	https://www.gsea-msigdb.org/gsea/msigdb
GraphPad Prism version 6.0 and version 9.5.1	GraphPad by Dotmatics	https://www.graphpad.com/updates

**Other**		

UCN2 transcript expression in human skin	Human protein atlas database	https://www.proteinatlas.org/ENSG00000145040-UCN2/single+cell+type/skin

### Resource availability

#### Lead contact

Further information should be directed to and will be fulfilled by the Lead Contact, Dr Niwa Ali (niwa.ali@kcl.ac.uk).

#### Materials availability

This study did not generate new unique reagents.

### Experimental model and study participant details

#### Human samples

This study involving the use of human participants was specifically approved by the institutional review board of Guy's Hospital (Guy's Research Ethics Committee, Ethics Committee Code: 06/Q0704/18) or St Thomas’ Hospital, London, UK Ethics Committee (Ref: 09/H0802/98) and conducted in accordance with the Helsinki Declaration. Informed written consent was obtained from all patients and healthy controls prior to enrolment into the study. Discarded skin was obtained from routine plastic surgery of healthy individuals.

#### Immunodeficient mice

Male and female NOD/scid/*IL-2Rγ*^*-/-*^ mice (NOD.cg-Prkdc^scid^Il2rg^tm1Wjl^/SzJ, abbreviated to NSG, obtained from The Jackson Laboratory) were used between 6-12 weeks of age. Mice were maintained under specific pathogen-free conditions and handled in accordance with the Institutional Committees on Animal Welfare of the United Kingdom Home Office (the Home Office Animals Scientific Procedures Act, 1986).

#### Monocyte-derived DCs (moDCs), T-cell isolation and culturing

Monocytes were isolated directly from whole blood or leukocyte cones using Rosettesep™ Human Monocyte Enrichment Cocktail (Stem Cell) according to manufacturer’s instructions, and cultured in complete RPMI medium supplemented with 1 % single donor plasma (NHS Blood and Transplant Tooting, London), 50 IU/mL Penicillin/ streptomycin (Gibco), 500 IU/mL GM-CSF (Peprotech) and 500 IU/mL IL-4 (R&D system). Fresh GM-CSF and IL-4 was added on day 2 and day 5 of a culture period of 7 days. CD141^+^ DDC-equivalent DCs were induced by the addition of 100 nM 1,25(OH)_2_D_3_ (VitD3; Sigma, dissolved in 100% Ethanol) on day 5 of moDC differentiation. Where indicated, VitD3 induced CD141^hi^ and CD141^lo^ DCs were stained 20min on ice with CD141-APC antibody (miltenyi Biotech) in RPMI 1640 medium supplemented with 10% fetal bone serum (FBS, Gibco), before resuspended in RPMI (without phenol red) supplemented with 2% FBS (Gibco), 2mM EDTA (Invitrogen) and 25mM HEPES (Gibco). Cells were sorted as shown in Figures S1B and S1C, before being used in co-culturing or qPCR. To study allostimulatory capacity of DCs, allogeneic CD4^+^ T-cells were prepared from healthy donor blood using RosetteSep human CD4^+^ T-cell enrichment cocktail (StemCell) according to manufacturers’ instructions. 5x10^5^ CD4^+^ T-cells were cultured in sterile polypropylene 5ml FACS tubes (BD) with 5x10^4^ allogeneic CD141^+^, CD1c^+^ DDCs (5x10^4^ cells/tube), VitD3-induced CD141^hi^ or CD141^lo^ DCs. Proliferation of alloreactive T-cells was assessed by [H^3^] thymidine incorporation (1 μCi/well, Amersham Bioscience) during the last 18 hours of 5 day cultures. In some circumstances, due to limiting DDC numbers, responder T-cells were scaled down accordingly resulting in lower thymidine counts. Where indicated, 10^-8^ M Urocortin 2 or 10^-6^ M anti-sauvagine-30 (anti-CRHR2 antagonist; aCRHR2; Phoenix Pharmaceuticals) was added at the beginning of the culture. DDC-induced CD4^+^CD25^hi^ T-cells were isolated by cell sorting CD11c^-^CD4^+^CD25^hi^ T-cells from DDC co-cultures and rested overnight. To assess *in vitro* suppressive capacity, 2x10^3^ sorted CD25^hi^ T-cells were co-cultured with 2x10^4^ autologous CD4^+^CD25^-^ effector T-cells (isolated from CD4^+^ T cells using Miltenyi CD25 microbead II) for 5 days in the presence of CD3/CD28 T-cell Expander Dynal Beads (Invitrogen). Cell proliferation was measured as described above. For Urocortin2-CD4^+^ T cell cultures, 10^-6^M Urocortin2 (Bachem) was supplemented to isolated CD4 T cells in the presence CD3/CD28 T-cell Expander Dynal Beads (1:1 bead to cell ratio according to manufacturer’s instructions, Invitrogen), and cultivated *in vitro* for 5 days before staining for flow cytometry and RNA isolation for quantitative PCR.

### Method details

#### Isolation of DDCs

DDC isolation was performed as previously described.^56^ In brief, skin was sliced into long strips followed by 5 mg/mL dispase digestion (StemCell) for 2 hours at 37°C. Epidermis and connective tissues in the lower dermis was removed and dermis was further sliced into 1-2 mm strips and then cultured in RPMI 1640, supplemented with 50 IU/mL penicillin, 50 μg/mL streptomycin, 2 mM L-glutamine (all from Invitrogen) and 10 % human AB serum (HS) (Sigma-Aldrich). Non-plastic adherent cells that had migrated out of dermis 48-72 hours after culture were harvested for flow cytometric analysis. Cell sorting of DDC subsets was performed by staining dermal cells and sorted with a FACS Aria II cell sorter (BD). Full gating strategy is shown in Figure S1A.

#### Flow cytometry staining

The following antibodies were used in different combinations: CD3-FITC (eBioscience); CD19-FITC, CD20-FITC, CD56-FITC, CD25-PE, CCR4-PECy7, CCR6-PerCp/Cy5.5,HLA-ABC-PE, CD3 - BUV395 (BD); CLEC9A-PE, CD1c-PE, CD1c-APC, CD141-PE,CD141-APC, CD278 (ICOS)-VioGreen, CD127 – APC, CD25 -PE and CD45 -VioBright515 (Miltenyi); CD3-FITC, PD-L1-PE and CD45-PECy7 (eBioscience); CD11c-PerCp/Cy5.5, CD4 -BV785, CD8a - BV650, CTLA-4 - BV605, FoxP3 - BV421 and Ki-67 - BV711 (BioLegend); CCR10-PE (R&D Systems); CD14-FITC, CD83-FITC, CD86-FITC, CD40-FITC and HLA-DR-PE (Invitrogen). Samples were stained in indicated antibodies combination for 20min on ice, washed with PBS supplemented with 15% (v/v) FBS, and acquired with a BD FACS Canto (BD) or BD LSRFortessa Cell Analyzer (BD) and data were analyzed by FlowJo (TreeStar) software.

#### Humanized mouse model of skin inflammation

Human skin samples were first keratomed using a hand-held air-powered dermatome (Zimmer) to obtain 500-700 μm split-thickness explants, consisting of the epidermis and superficial dermis. Explants were kept at 4°C in RPMI 1640 supplemented with 50 IU/mL penicillin, 50 μg/mL streptomycin, 2 mM L-glutamine and subsequently cut into 1-1.5 cm^2^ pieces and transplanted orthotopically onto the backs of NSG mice, as previously described.^19^ NSG mice bearing human skin transplants were allowed to engraft for 5-6 weeks before adoptive transfer of human cells. Purified anti-mouse Gr1 mAb (100 μg; BioXell) was injected intraperitoneally on the day of skin grafting and then 4 subsequent injections every 4 days to deplete mouse granulocytes during the early post-transplant period. Following adequate skin engraftment, 5 x 10^6^ allogeneic (to the skin) CD4^+^ T-cells depleted of CD25^+^ cells were adoptively transferred intravenously via the tail vein to induce alloimmune skin inflammation, as published.^33^ Where indicated, 500ng Urocortin 2, 500ng aCRHR2, or PBS was injected in a total volume of 30 μl directly into the human skin transplant every 5 days for 15 days and skin grafts harvested on day 18 for immunohistological assessment for markers of skin inflammation.

#### Immunofluorescence staining

Laser confocal microscopy was performed (TCS SP2; Leica). The following antibodies were used to stain OCT embedded cryosections: CD141 (Miltenyi); CD3 (Dako); Ki-67 (Abcam); Urocortin 2 (Phoenix Pharmaceuticals); FoxP3 (BD); goat anti-mouse IgG AlexaFluor555 or AlexaFluor488 and goat anti-rabbit IgG AlexaFluor555 or AlexaFluor488 (Invitrogen). ProLong Gold antifade reagent with DAPI (Invitrogen) was used for nuclear staining.

#### Determination of IL-10 production

2 x 10^4^ DCs were cultured in 96-well flat-bottom plates with 100 μL of complete RPMI medium containing 10 % human AB serum (HS) for 2 days in the absence or presence of CD40 ligand-transfected cells (CD40L Tx, 5 x 10^4^ cells/well). CD40L Tx was kindly provided by Prof Giovanna Lombardi.^57^ IL-10 levels in the supernatant were assayed by using the MILLIPLEX MAP Human Cytokine Kit (Millipore) and acquired on a Luminex 100 flow-based sorting and detection analyzer (Luminex Corporation).

#### RNA extraction and quantitative RT-PCR (qRT-PCR)

RNA extraction was performed using NucleoSpin RNA XS Kit (Macherey-Nagel GmbH & Co) according to manufacturer’s instructions, and retro transcribed into cDNA using Superscript II Reverse Transcriptase (invitrogen) or iScript (BioRad). Human UCN2, LIPG, PVALB, FOXP3 and CTLA4 expression was assessed by multiplex real-time quantitative RT-PCR using Taqman assays (Applied Biosystems) according to manufacturers’ instructions. For each sample, mRNA abundance was normalized to the amount of human GAPDH. Data analysis was performed using the ΔΔCt method: results were expressed as relative mRNA levels or as fold change in arbitrary units.

#### RNA microarray experiments

RNA integrity was assessed prior to performing expression arrays. The RNA Nano kits (Agilent Technologies) were used with the Agilent 2100 Bioanalyzer to quantify and analyze the integrity of total RNA. The RIN (RNA Integrity Number) was set at 9.0 and any sample that did not meet this threshold was excluded from RNA microarray analyzes. Total RNA, extracted as above, was firstly amplified with TargetAmp™-Nano Labeling Kit for Illumina® Expression BeadChip® (epicentre). 500ng of total RNA was then used with Human Ref-6 Expression BeadChips (Illumina, Ambion) to generate RNA expression data as per manufacturer’s instructions. All were scanned with a Bead Array Reader (Illumina).

#### Differential gene expression analysis

Differential Expression Analysis was carried out using the MATLAB software and functions from Bioinformatics Toolbox (http://www.mathworks.com/help/toolbox/bioinfo/). Genes that were not expressed or had only small variability across the samples were removed. T-test was conducted to identify significant changes for each gene in their expression values between the different phenotypes.^58^ Genes with p-value < 0.05 and fold change (fch) > 1.5 were considered to be statistically significant. Differentially expressed genes were further analyzed for enriched Gene Ontology (GO) terms and KEGG pathways using the DAVID 6.7 (http://david.abcc.ncifcrf.gov/) bioinformatics database.^59^ Biological terms that have many genes in common were grouped into a module of related terms and genes.

#### Pathway activity and *Z* score

Pathway-specific gene expression profiles were built from microarray measurements using pathway-gene associations from KEGG database^60^ and data from the C2 functional set were downloaded from the Molecular Signature Database (MSigDB database v3.0, downloaded in September 2010). Expression profiles for each (S) sample obtained across *G genes* are summarized by a *S x G* matrix, where each entry denotes the expression level of gene *g* in sample *s*. Further, given a pathway and expression values of all its member genes we calculated pathway activity, as published.^61^ Expression values *gij* are normalized to z-transformed scores *zij*, were averaged into a combined *z*-score and were used to calculate the activity *aj* (to stabilize the variance of the mean we used the square root of the number of genes per pathway as the denominator in the calculation). According to the pathway activities, a new expression matrix is generated with pathways (P) as features and genes (G) as attributes. The generated pathway activity matrix was then input into support vector machines (SVM) classifier to rank the pathways that best discriminate the samples. The pathway analysis strategy is shown in Figure S3.

#### Ultraviolet radiation human study

Full details of the irradiation protocol are given by.^29^ Briefly, previously unexposed buttock skin was exposd to 50 J/cm^2^ UVA1 and 30 mJ/cm^2^ monochromatic UVB (300nm). These doses are approximately 1 MED. 4 mm punch biopsies were taken at 6 and 24 hours post irradiation and processed for immunohistology, as outlined above. The details of the three healthy young sun-sensitive skin type I/II^62^ volunteers are shown in Table 1.

### Quantification and statistical analysis

Statistical analysis was performed using GraphPad Prism version 6.0 (GraphPad Software). Results were assessed for normal Gaussian distribution and then analyzed by unpaired t test, one-way ANOVA test, or two-way ANOVA as appropriate. For markers of skin inflammation quantification, at least one independent image was acquired from each individual and two independent researchers blinded from treatment groups counted every image. Staining intensity quantification of healthy volunteer irradiated skin was performed using NIS elements BRv3 software package. Individual CD141^+^ DDCs or total dermis were gated and the intensity of red (CD141) or green staining (UCN2) calculated. Values of P<0.05 were considered significant.

## Data Availability

• Microarray data are deposited at GEO and are publicly available. • Accession numbers are listed in the key resources table. • This paper does not report original code.

## Supplemental information

Document S1. Figures S1‒S6

Table S1. List of differentially expressed genes in the pairwise analysis of CD141^+^ DDCs/CD1c^+^ DDCs, related to Figure 2

Table S2. List of differentially expressed genes in the pairwise analysis of CD141^hi^ VitD3 moDCs/moDCs, related to Figure 2
