# Supplementary material for: Human skin CD141+ dendritic cells regulate cutaneous immunity via the neuropeptide urocortin 2
Source: iScience. 2023 Sep 26;26(10):108029. doi: 10.1016/j.isci.2023.108029 (PMC10583083; doi:10.1016/j.isci.2023.108029)
Supplement: Document S1. Figures S1‒S6 [file mmc1.pdf]

iScience, Volume *26*

Supplemental information

Human skin CD141^{+} dendritic cells regulate cutaneous immunity via the neuropeptide urocortin 2

Prudence PokWai Lui, Chrysanthi Ainali, Chung-Ching Chu, Manuela Terranova-Barberio, Panagiotis Karagiannis, Angela Tewari, Niloufar Safinia, Ehsan Sharif-Paghaleh, Sophia Tsoka, Grzegorz Woszczek, Paola Di Meglio, Giovanna Lombardi, Antony R. Young, Frank O. Nestle, and Niwa Ali

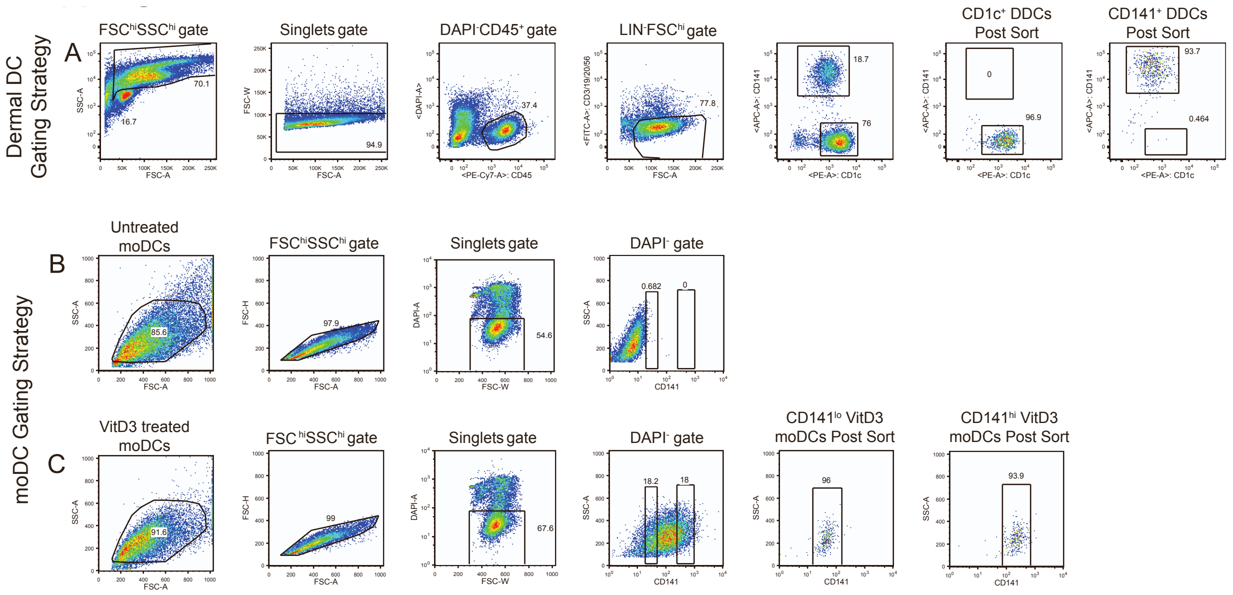

Figure S1. Skin DDC and VitD3 moDC gating and sorting strategies, related to Figure 1. (A) Cells obtained from healthy human dermis were stained for DAPI (dead cells), CD45, Lineage cocktail (CD3, CD19, CD20, CD56), CD1c and CD141. CD141^{+} DDCs were sorted as CD141^{hi}CD1c^{lo} and CD1c^{+} DDCs as CD1c^{hi}CD141^{-} (shown in red) (B) Monocyte derived DCs (moDCs) were stained for DAPI (dead cells) and CD141. moDCs were sorted as DAPI^{-} cells and (C) VitD3 moDCs were sorted as CD141^{lo} or CD141^{hi}, usually constituting the lower and upper ~18% of CD141^{+} cells. All results are representative of greater than twenty independent experiments from different skin and blood donors.

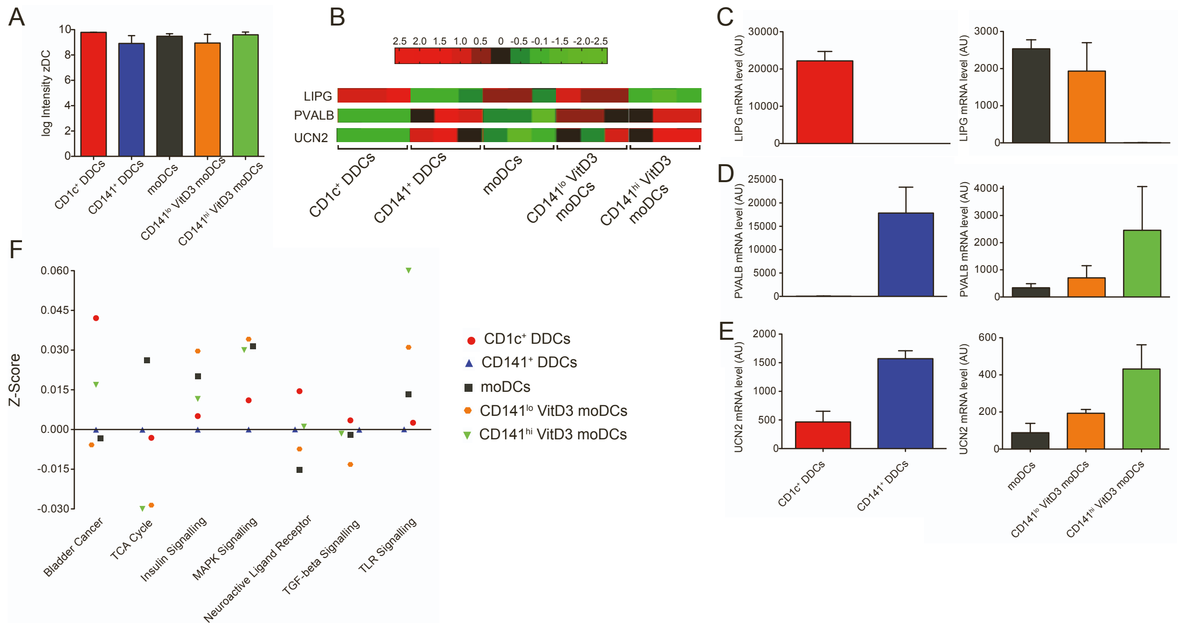

Figure S2. Skin DDC and moDC subset pathway activity and gene expression validation. related to Figure 2. (A) Raw signal intensity of zbtb46 (zDC) from the gene expression array. (B) Heat map of three highly differentially expressed genes LIPG, PVALB, and UCN2, (C-E) validated by quantitative PCR. (F) Pathway activity (z-score) of DDC and moDC subsets with CD141^{+} DDCs as the reference DC subset (set as 0). All data are combined from three to five human skin and blood donors, from two independent experiments.

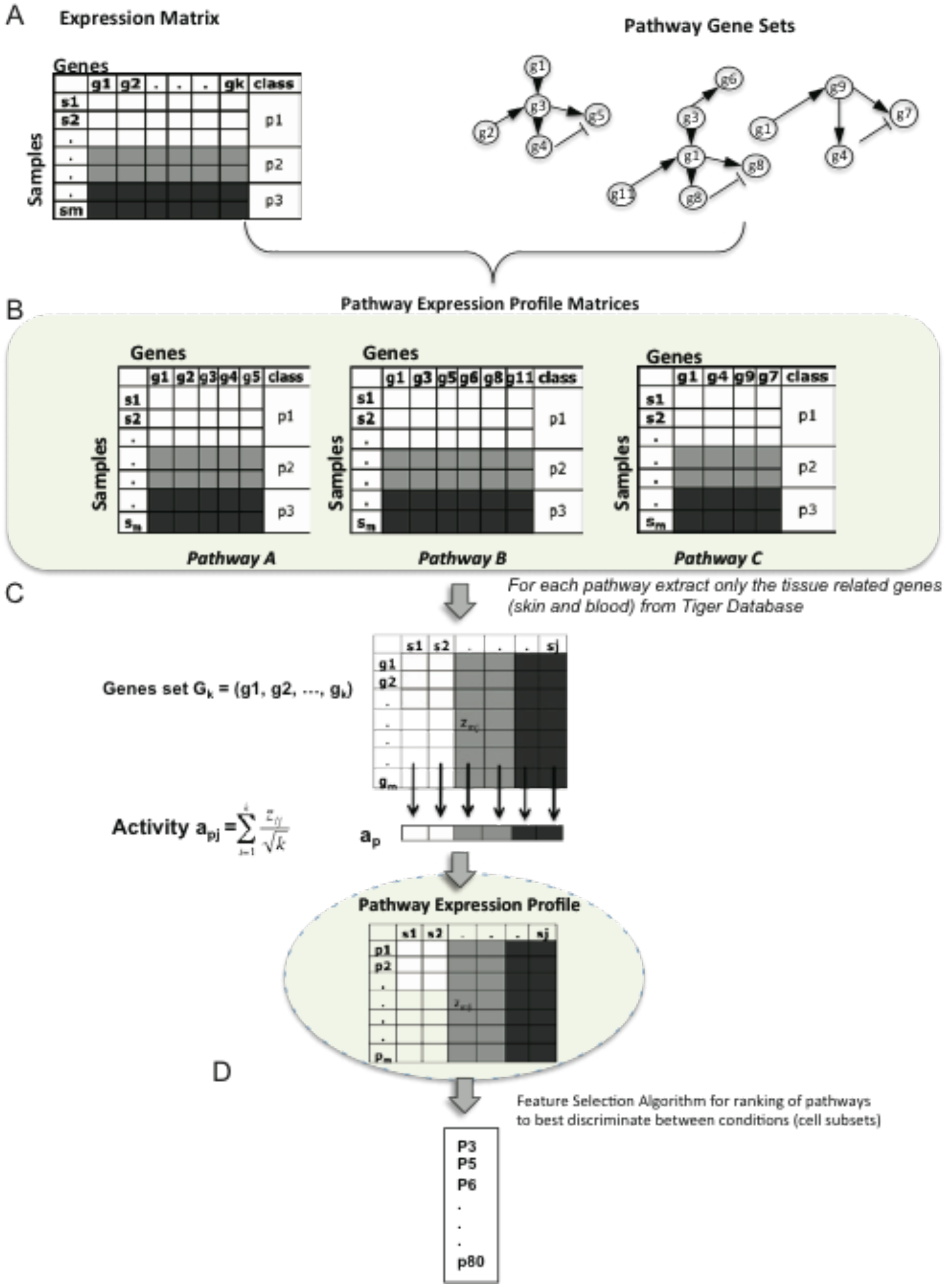

Figure S3. Schematic representation of the computational model for pathway activity analysis, related to STAR Methods. (A) The gene expression matrix for disease tissue samples, together with gene-to-pathway correspondence according to KEGG biochemical pathways were used as an input and their integration generated (B) pathway-specific gene expression profiles. (C) For each pathway matrix, only tissue related genes from Tiger Database were extracted to calculate the pathway activities (as described in the materials and methods). (D) The pathway expression profiles were then ranked to best discriminate the samples.

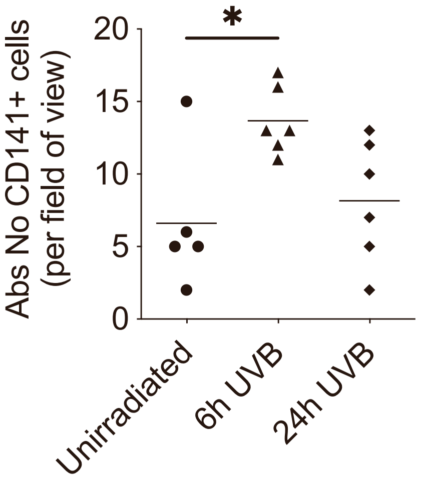

Figure S4. UVB exposure of human skin increases CD141+ Skin DC abundance, related to Figure 3. Human skin was biopsied from healthy volunteers prior to irradiation and at 6 and 24 hours post irradiation with 1 MED of 30 mJ/cm2 solar UVB and assessed via immunofluorescence for CD141. The absolute number of dermal CD141+ DCs were quantified per field of view. Data are combined from three healthy volunteers in total. One-way ANOVA, *P<0.05.

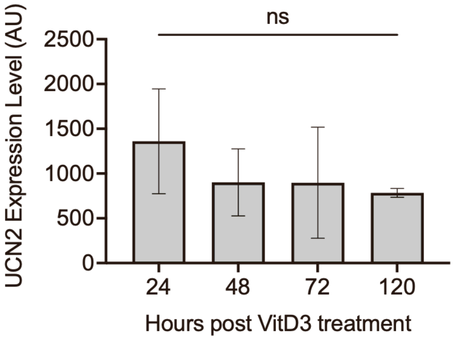

Figure S5. CD141high moDCs sustain UCN2 expression, related to Figure 3. CD141hi VitD3 moDCs were FACS sorted at time intervals post VitD3 treatment and assessed for UCN2 expression via qPCR. Error bars indicate SEM. Data are combined from three to five human blood donors from two independent experiments. One-way ANOVA. ns = not significant.

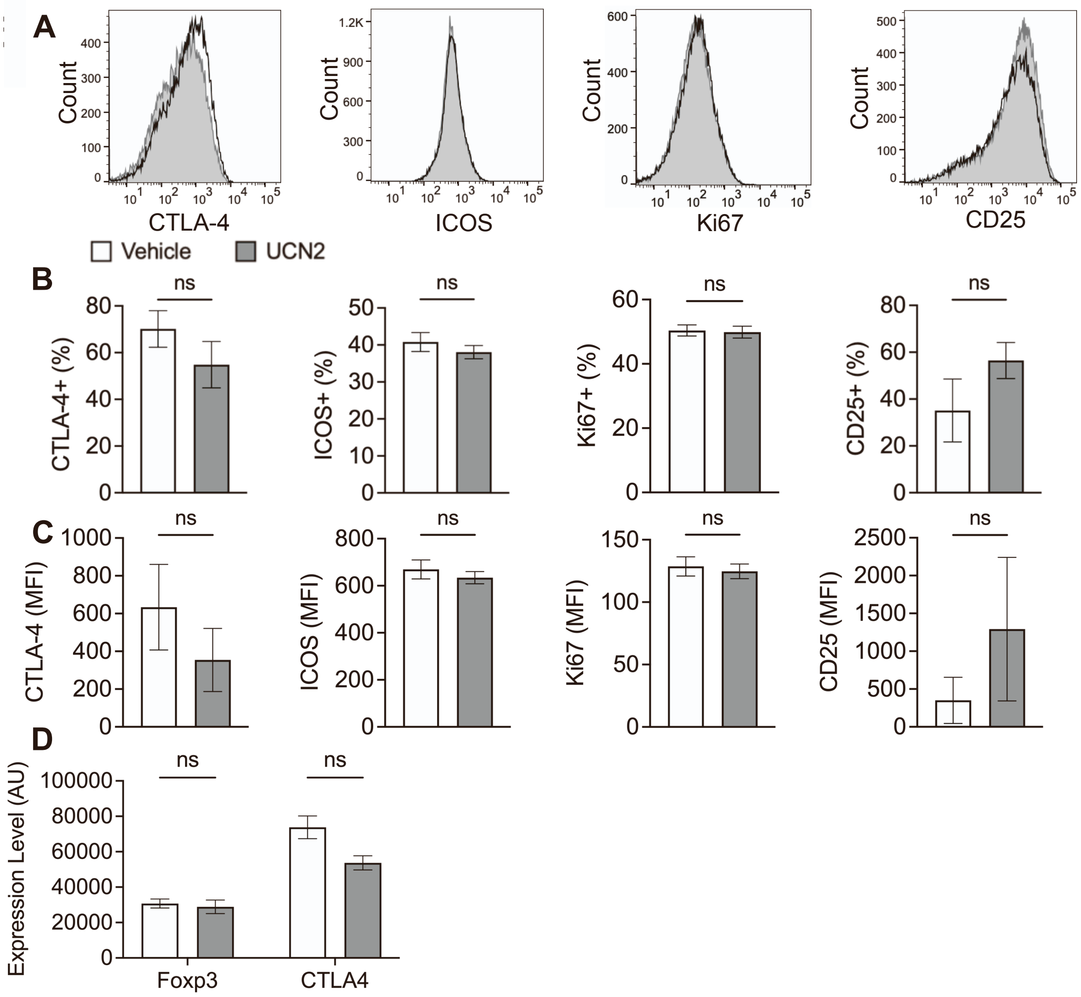

Figure S6. UCN2 alone is not sufficient to drive the expression of Treg associated markers in CD4+ T cells, related to Figure 4. CD4+ T-cells were cultured in the presence of vehicle control or UCN2 peptide for 5 days. Cells were then stained via flow cytometry for (A) CTLA-4, ICOS, Ki67, and CD25. Summary data for (B) frequency and (C) median fluorescence intensity (MFI) expression, and (D) Transcript expression of FoxP3 and CTLA-4 as measured by qRT-PCR. Results are (A) representative of and (B-D) combined from two independent experiments from three healthy volunteers in total. (B-C) Unpaired students t test, (D) Two-way ANOVA. ns = not significant.
